# The potential roles of m^6^A modification in regulating the inflammatory response in microglia

**DOI:** 10.1186/s12974-021-02205-z

**Published:** 2021-07-05

**Authors:** Qi Li, Shaohong Wen, Weizhen Ye, Shunying Zhao, Xiangrong Liu

**Affiliations:** China National Clinical Research Center for Neurological Diseases, Beijing Tiantan Hospital, Capital Medical University, 119 South Fourth Ring West Road, Fengtai District, Beijing, 100070 People’s Republic of China

**Keywords:** m^6^A mRNA, m^6^A lncRNA, Microglia, Polarization, Phenotype, Inflammatory response, Microarray, Methylated m^6^A RNA immunoprecipitation, Bioinformatics analysis

## Abstract

**Background:**

Microglia are key regulators of the inflammatory response in the brain. Adenosine in RNAs can be converted to m^6^A (N^6^-methyladenosine), which regulates RNA metabolism and functions as a key epitranscriptomic modification. The m^6^A modification pattern and m^6^A-related signatures under pro-inflammatory and anti-inflammatory conditions of microglia remain unclear.

**Methods:**

Primary rat microglia were differentiated into pro-inflammatory M1-like (M1-L), anti-inflammatory M2-like (M2-L), and resting, unstimulated (M0-L) phenotypes. m^6^A mRNA and lncRNA epitranscriptomic microarray analyses were performed, and pathway analysis was conducted to understand the functional implications of m^6^A methylation in mRNAs and lncRNAs. The m^6^A methylation level and gene expression of mRNAs and lncRNAs were subsequently verified by m^6^A Me-RIP and qRT-PCR.

**Results:**

A total of 1588 mRNAs and 340 lncRNAs, 315 mRNAs and 38 lncRNAs, and 521 mRNAs and 244 lncRNAs were differentially m^6^A methylated between M1-L and M0-L (M1-L/M0-L), M2-L and M0-L (M2-L/M0-L), M2-L and M1-L (M2-L/M1-L), respectively. Furthermore, 4902 mRNAs, 4676 mRNAs, and 5095 mRNAs were identified distinctively expressed in M1-L/M0-L, M2-L/M0-L, and M2-L/M1-L, respectively. Pathway analysis of differentially m^6^A methylated mRNAs and lncRNAs in M1-L/M0-L identified immune system, signal transduction, and protein degradation processes. In contrast, the distinct m^6^A methylated mRNAs in M2-L/M0-L were involved in genetic information processing, metabolism, cellular processes, and neurodegenerative disease-related pathways. We validated m^6^A methylation and the expression levels of five mRNAs and five lncRNAs, which were involved in upregulated pathways in M1-L/M0-L, and five mRNAs involved in upregulated pathways in M2-L/M0-L.

**Conclusions:**

These findings identify a distinct m^6^A epitranscriptome in microglia, and which may serve as novel and useful regulator during pro-inflammatory and anti-inflammatory response of microglia.

**Supplementary Information:**

The online version contains supplementary material available at 10.1186/s12974-021-02205-z.

## Introduction

Microglia are non-neuronal cells, which belong to the glial population of central nervous system (CNS) cells. As the resident immune cells of the brain parenchyma, microglia act as central communicators between the nervous and immune systems to coordinate homeostatic and immune surveillance functions of the CNS [1, 2]. Stimulated by pathogens, injuries, or pathological stresses, the homeostatic microglia (M0-L) can functionally reprogram [3, 4]. Similar to macrophages, microglia adopt a “classical” or an “alternative” activation phenotype under defined environmental stimuli [5]. Classically activated microglia (M1-L) have been associated with anti-microbial activity through the production of pro-inflammatory mediators, whereas the alternatively activated microglia (M2-L) have been related to tissue repair and homeostasis restoration [6–9].

The Immunological Genome (ImmGen) Project, which was the first systematic study of the expression profiles of murine macrophages from different organs, revealed a high diversity among different tissue-resident macrophage populations, suggesting flexibility to adapt to their environment [6]. The comparison of microglial gene expression profiles with the transcriptomes of other peripheral immune cells demonstrated that microglial are unique within the innate immune cell repertoire [5]. In addition, the comparison of microglial gene expression profiles with the transcriptome of other brain cells revealed that microglia are distinct from other CNS cell populations [10].

After sensing disruption in CNS homeostasis, microglia rapidly change their gene expression programs and functional profiles. Recent genome-wide transcriptional studies revealed a unique molecular signature selectively expressed in homeostatic microglia [5, 7, 11, 12] that is lost in disease and during ageing [13–17]. Moreover, single-cell transcriptomics revealed that microglia isolated from lipopolysaccharide (LPS)-injected mice display a global downregulation of their homeostatic signature together with an upregulation of inflammatory genes [18].

Among the mechanisms that establish RNA expression profiles in response to environmental influences, regulation of the chromatin landscape, including epigenetic histone and DNA modifications, enhancers, and transcription factors, has been shown to be critical [19, 20]. However, more recently, RNA modifications are emerging as important regulatory mechanisms in gene expression. Among the over 150 RNA modifications, N^6^-methyladenosine (m^6^A) RNA methylation is the predominant form, occupying approximately 0.3% of total adenosine residues [21–23]. At the molecular level, m^6^A is known to affect RNA stability, translation, microRNA biogenesis, splicing, X-chromosome inactivation, and other biological processes. In recent years, various biological phenomena have been associated with the m^6^A modification on RNA, such as obesity, cancer, cell fate transitions, fertilization, and pluripotency.

In this study, we describe the transcriptome-wide profile of m^6^A in mRNAs and long non-coding RNAs (lncRNAs) in M0-L, M1-L, and M2-L microglia. Furthermore, bioinformatic analysis reveals a potential interaction between RNA m^6^A modifications and signal transduction, immune system processes, genetic information processing, metabolism, cellular processes, and neurodegenerative disease signaling pathways in microglia.

## Methods

### Primary newborn rat microglia culture

Primary microglia were isolated from newborn (< 24 h) Sprague-Dawley (SD) rat brains as previously described [24]. Brains were dissected, and the cerebellum, brainstem, and olfactory bulb were removed and placed on ice. Subsequently, meninges and large blood vessels were carefully stripped, and brains were minced in cold Hank’s Balanced Salt Solution buffer. Tissue dissociation was performed by trypsinization for 10 min (0.05% Trypsin–EDTA; Gibco, Life Technologies, Gaithersburg, MD, USA). Cells were resuspended in FBS (Fetal bovine serum, Gibco) and the trypsin was neutralized by FBS. The cellular suspension was centrifuged and resuspended in complete medium DMEM/F12 (Gibco) supplemented with 10% FBS and 100 U/ml penicillin/streptomycin (Life Technologies). After filtration, cells were seeded into cell culture flasks (T75) and allowed to attach and grow at 37 °C in a humidified atmosphere containing 5% CO_2_. The culture media was renewed after 12 h, 3 days, 5 days, 7 days, 9 days, 11 days, and 13 days of culture. On day 14, microglia were shaken at 200 rpm for 60 min, collected, and plated in 24-well plates at a density of 2 × 10^5^/well. Microglia were activated with different compounds for 48 h. For M1-L induction, lipopolysaccharide (LPS, 100 ng/mL, Sigma, St. Louis, MO, USA) and interferon-γ (IFNγ, 20 ng/mL, Peprotech, Rocky Hill, NJ, USA) were added to the cultures. For M2-L induction, interleukin (IL)-4 (20 ng/mL, Peprotech), and IL-13 (20 ng/mL, Peprotech) were added daily.

### RNA isolation and methylated RNA immunoprecipitation (IP)

Total RNA was isolated with Trizol (Invitrogen, Carlsbad, CA, USA). The purity and amount of RNA were determined using NanoDrop ND-1000. A total of 3 μg of RNA and m^6^A spike-in control mixture were added to the IP buffer (50 mM Tris-HCl, pH 7.4, 150 mM NaCl, 0.1% NP40, 40 U/μL RNase inhibitor) and incubated with 2 μg anti-m^6^A rabbit polyclonal antibody (202003, Synaptic Systems, Goettingen, Germany) at 4 °C for 2 h. Dynabeads™ M-280 Sheep Anti-Rabbit IgG (11203D, Invitrogen) were blocked with 0.5% BSA at 4 °C for 2 h, washed, and resuspended in the total RNA-antibody mixture prepared above, and incubated at 4 °C for a further 2 h. The modified RNA was eluted with elution buffer (10 mM Tris-HCl, pH 7.4, 1 mM EDTA, 0.05% SDS, 40 U Proteinase K) at 50 °C for 1 h, and the unmodified RNA was recovered from the supernatant. The modified RNA and unmodified RNA were extracted by acid phenol-chloroform and ethanol precipitation, respectively.

### Microarray hybridization

The modified RNA and unmodified RNA were added to an equal amount of calibration spike-in control RNA, amplified, and labeled with Cy5 and Cy3 fluorescent dye, respectively, as cRNA using the Arraystar Super RNA Labeling Kit (Arraystar, MD, USA) and purified by the RNeasy Mini Kit (Qiagen, Hilden, Germany). The Cy3- and Cy5-labeled cRNA were mixed and hybridized to a rat m^6^A mRNA and lncRNA Epitranscriptomic Microarray (4 × 44 K, Arraystar) that contained 27,770 mRNA and 10,582 lncRNA. The hybridized arrays were washed, fixed, and scanned in two-color channels using an Agilent Scanner G2505C.

### Microarray data analysis

Agilent Feature Extraction software (11.0.1.1) was used to analyze the acquired array images. Raw intensities of Cy5-labeled RNA and Cy3-labeled RNA were normalized with an average of log_2_-scaled spike-in RNA control. The m^6^A methylation level was calculated for the percentage of modification based on the Cy5-labeled and Cy3-labeled normalized intensities. The fold change and *P* values were calculated for each transcript between two comparison groups. Differentially m^6^A-methylated RNAs between two comparison groups were identified by filtering with the fold change and two-tailed Student’s *t* test. Fold-change (FC) ≥ 1.5 or ≤ 0.7, and *P* < 0.05 were used to identify the differentially m^6^A methylated RNAs in M1-L/M0-L, M2-L/M0-L, and M2-L/M1-L. Hierarchical clustering was performed to show the differential m^6^A-methylation pattern among samples.

### Pathway analysis

Pathway analysis was used to identify the pathways associated with differentially expressed genes according to Kyoto Encyclopedia of Genes and Genomes (KEGG) (http://www.genome.jp/kegg/). Differentially expressed genes were mapped to defined KEGG pathways. Enrichment scoring, which is equal to −log10 (*P* value), was used to measure pathway significance and specificity.

### Methylated RNA immunoprecipitation (MeRIP)

MeRIP assay was performed with the Magna MeRIP™ m^6^A Kit (Millipore, Billerica, MA, USA) according to the manufacturer’s protocol. Total RNA was fragmented into 100 nucleotides followed by magnetic IP with a monoclonal antibody against m^6^A. Immunoprecipitated RNA was analyzed by qRT-PCR normalized with the input RNA, and each experiment was repeated three times independently. The sequences of the primers in reference to the m^6^A motif regions of the five mRNAs and five lncRNAs are listed in Additional file 1.

### Quantitative real-time polymerase chain reaction (qRT-PCR)

qRT-PCR was used to confirm the expression of mRNAs and lncRNAs in microglia. First-strand cDNA was synthesized from total RNA (1 μg) using the SuperScript III First Strand Synthesis Super System (Invitrogen). qRT-PCR was performed on an ABI QuantStudio3 system (Applied Biosystems, Foster City, CA, USA) using the RT^2^ SYBR Green ROX FAST Mastermix (Qiagen). The amplification conditions were 95 °C for 10 min, then 40 cycles at 95 °C for 15 s and 60 °C for 1 min. All of the primers used are listed in Additional file 2. Gene expression was normalized to GAPDH mRNA. Expression was calculated using the 2^−∆∆CT^ method.

### Statistics

For pathway analysis, a two-sided Fisher’s exact test was used to classify the pathway, and *P* values were computed for the pathway of each of the differentially expressed genes. GraphPad Prism 8.0 software (GraphPad Software Inc., La Jolla, CA, USA) was used for qRT-PCR statistical analyses. These data were presented as mean ± standard error of the mean (SEM). Significant differences were assessed by Student’s t test for two-group comparisons. Statistical significance was set at *P* < 0.05.

## Results

### m^6^A modified transcripts are significantly altered in M0-L, M1-L, and M2-L microglia

To elucidate the effect of microglial phenotype on transcript-specific m^6^A changes, we profiled the immunoprecipitated m^6^A methylated RNAs isolated from the primary microglia subjected to treatment with LPS plus IFNγ (M1-L) and IL-4 plus IL-13 (M2-L). Untreated primary microglia served as the M0-L homeostatic phenotype.

The m^6^A methylated RNAs labeled with Cy5 fluorescent dye were analyzed using the rat m^6^A mRNA and lncRNA Epitranscriptomic Microarray (M0-L: n = 3, M1-L: n = 3; M2-L: n = 3) with probes for 38,352 RNAs (27,770 mRNAs and 10,582 lncRNAs). Microarray profiling showed that 1928 transcripts (1588 mRNAs and 340 lncRNAs), 353 transcripts (315 mRNAs and 38 lncRNAs), and 765 transcripts (521 mRNAs and 244 lncRNAs) were differentially m^6^A methylated in M1-L/M0-L, M2-L/M0-L, and M2-L/M1-L, respectively (fold change ≥ 1.5 or ≤ 0.7, *P* < 0.05; Additional files 3 and 4).

**Fig. 1:**
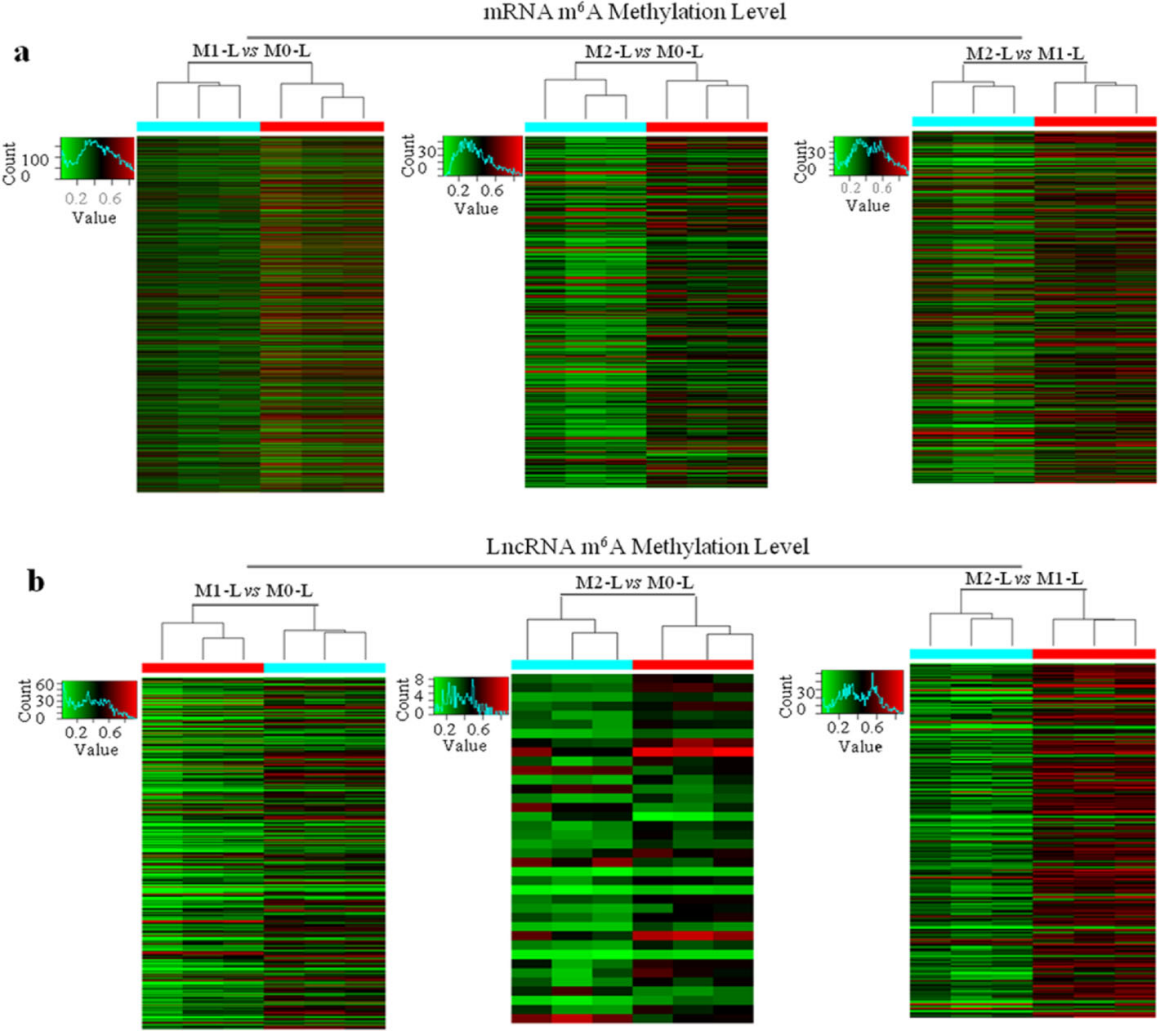
mRNA and lncRNA m^6^A modification profile changes in different primary rat microglia phenotypes. Hierarchical clustering of all samples revealed the non-random partitioning of samples into three major groups: M1-L vs M0-L; M2-L vs M0-L; and M2-L vs M1-L. Each column represents one sample and each row represents one mRNA (**A**) or lncRNA probe set (**B**)

In the M1-L/M0-L comparison, 543 mRNAs and 263 lncRNAs were hyper-methylated, while 1045 mRNAs and 77 lncRNAs were hypo-methylated. Most of the differentially methylated mRNAs (65.6%) were significantly hypo-methylated, while most of the lncRNAs (77.4%) were significantly hyper-methylated. In the M2-L/M0-L comparison, only 46 mRNAs and 7 lncRNAs were differentially hyper-methylated, while the majority of the mRNAs (269; 85.4%) and lncRNAs (31; 81.6%) were differentially hypo-methylated. Hierarchical clustering identified the inter-relationships of the samples and grouped them based on the similarities of the m^6^A methylation level (Fig. 1). These data demonstrate that M0-L, M1-L, and M2-L microglia display distinct m^6^A modified mRNA and lncRNA patterns, with the largest change in m^6^A methylation levels in the pro-inflammatory M1-L microglia.

### Identification of the gene expression profile associated with m^6^A methylation of mRNAs

**Fig. 2:**
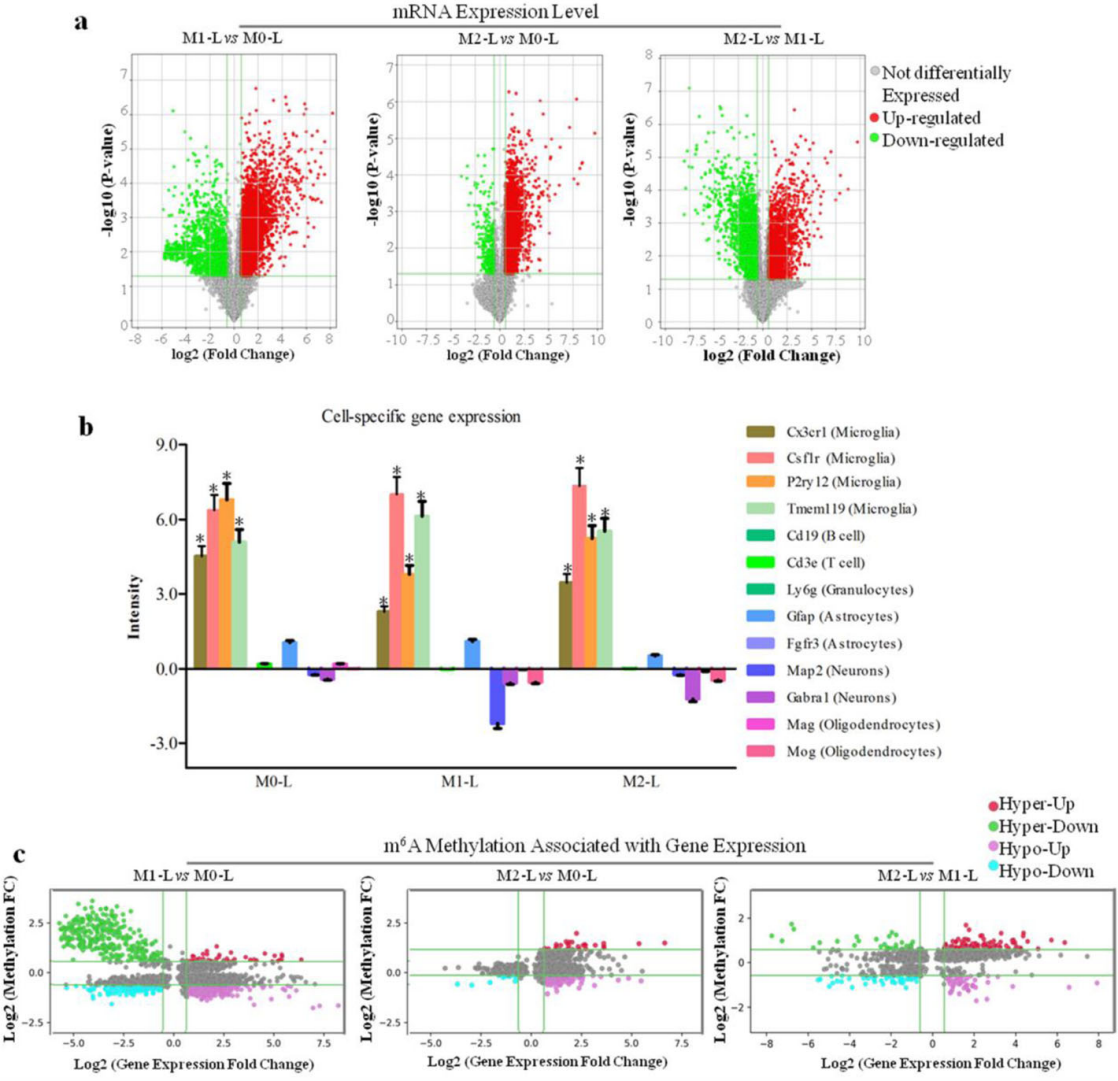
mRNA expression analysis of M0-L, M1-L, and M2-L phenotypes in microglia. **A** Volcano plot analysis of 3627 upregulated and 1275 downregulated mRNAs (M1-L vs M0-L, *P* < 0.05); 4360 upregulated and 316 downregulated mRNAs (M2-L vs M0-L, *P* < 0.05); 1896 upregulated and 3199 downregulated mRNAs (M2-L vs M1-L, *P* < 0.05). Red boxes represent ≥ 1.5-fold change difference, *P* < 0.05. Green boxes represent ≤ 0.7-fold change difference, *P* < 0.05. **B** Expression of markers specific to different brain and immune cell types in M0-L, M1-L, and M2-L samples. Data represent mean ± SD of three biological replicates. **C** Association analysis of m^6^A methylation and mRNA expression: M1-L vs M0-L: 39 Hyper-Up mRNAs, 319 Hyper-Down mRNAs, 515 Hypo-Up mRNAs, 138 Hypo-Down mRNAs (*P* < 0.05); M2-L vs M0-L: 26 Hyper-Up mRNAs, 0 Hyper-Down mRNAs, 177 Hypo-Up mRNAs, 11 Hypo-Down mRNAs (*P* < 0.05); M2-L vs M1-L: 95 Hyper-Up mRNAs, 32 Hyper-Down mRNAs, 48 Hypo-Up mRNAs, 49 Hypo-Down mRNAs (*P* < 0.05)

To investigate the mRNA expression patterns among M0-L, M1-L, and M2-L, we also profiled total mRNAs labeled with Cy3 fluorescent dye using above-mentioned microarray. A total of 4902 mRNAs were identified that had significantly differential expression in M1-L/M0-L, 4676 mRNAs showed differential expression in M2-L/M0-L, and 5095 mRNAs showed differential expression in M2-L/M1-L (Fig. 2A, Additional file 5). Compared with markers for other parts of the brain and immune cells, microglia-specific markers were detected at high levels in all samples tested, indicating a high purity of sorted microglia (Fig. 2B).

Next, the intersection of the differentially expressed mRNA and differentially methylated m^6^A mRNAs were selected, and the m^6^A modification-associated gene expression profiles in M1-L/M0-L, M2-L/M0-L, and M2-L/M1-L were obtained. Of these m^6^A modification-associated mRNAs, we found four modes of interaction including (i) m^6^A hyper-methylated and upregulated mRNAs; (ii) m^6^A hyper-methylated and downregulated mRNAs; (iii) m^6^A hypo-methylated and upregulated mRNAs, and (iv) m^6^A hypo-methylated and downregulated mRNAs (Fig. 2C, Additional file 6, 7, and 8). These results suggest that RNA m^6^A methylation could play key roles in the regulation of the stability and expression levels of mRNA in pro-inflammatory and anti-inflammatory microglia.

**Fig. 3:**
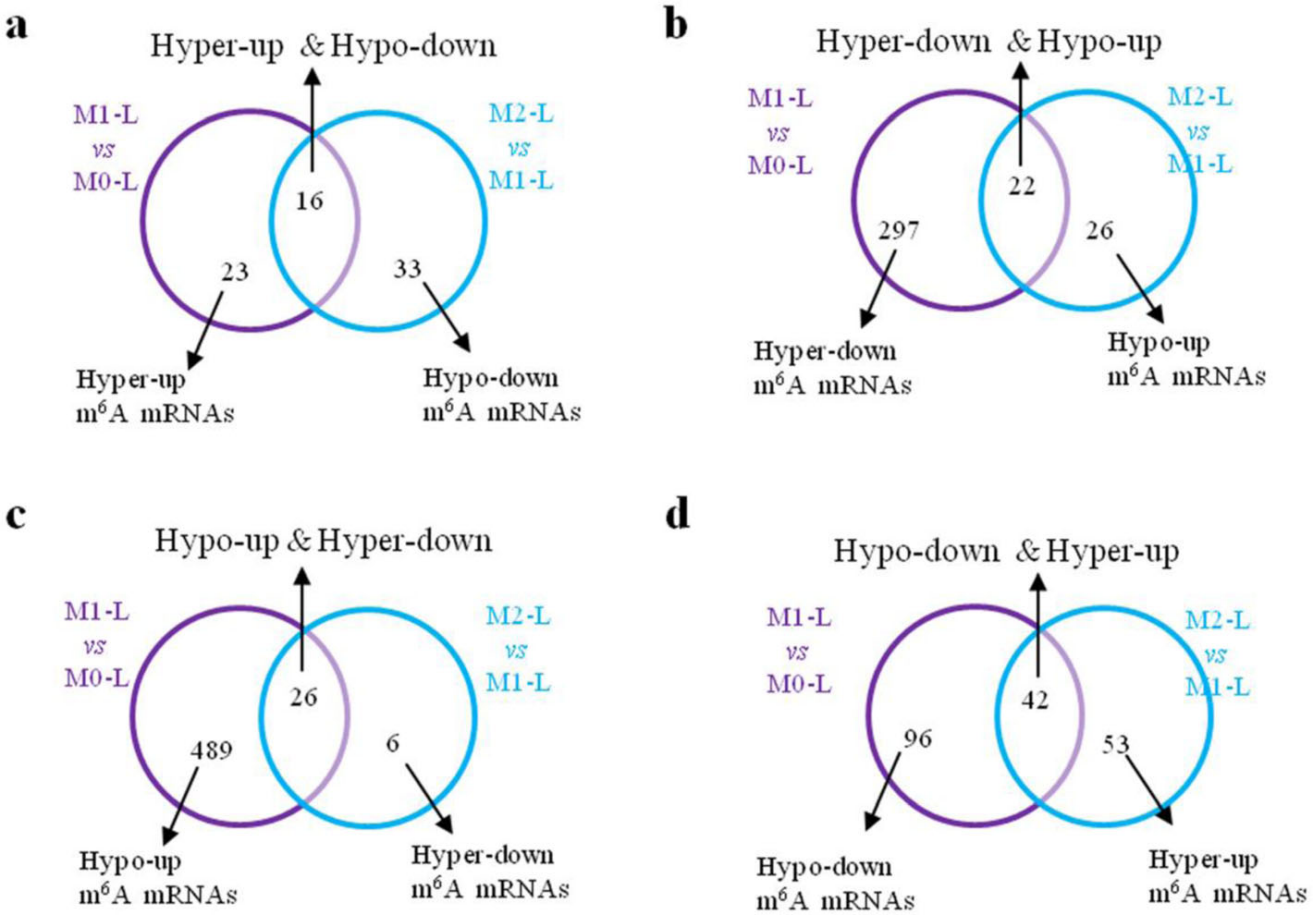
Expression patterns of m^6^A modification-associated genes among the M0-L, M1-L and M2-L phenotypes. Venn diagrams showing unique and common **A** Hyper-Up and Hypo-Down, **B** Hyper-Down and Hypo-Up, **C** Hypo-Up and Hyper-Down, **D** Hypo-Down and Hyper-Up genes between “M1-L vs M0-L” (purple) and “M2-L vs M1-L” (blue). (*P* < 0.05)

Furthermore, we analyzed the expression trends of these m^6^A modification-associated genes among the M0-L, M1-L, and M2-L phenotypes. As shown in Fig. 3, 41.0% of hyper-methylated/upregulated genes in M1-L/M0-L were hypo-methylated/downregulated in M2-L/M1-L, while 30.4% of hypo-methylated/downregulated genes in M1-L/M0-L were hyper-methylated/upregulated in M2-L/M1-L. We also found that only 5.0% of hypo-methylated/upregulated genes and 6.9% of hyper-methylated/downregulated genes in M1-L/M0-L are hyper-methylated/downregulated and hypo-methylated/upregulated in M2-L/M1-L, respectively. Strikingly, only a single gene (*Diaph3*) was hypo-methylated/downregulated in M1-L/M0-L and hyper-methylated/upregulated in M2-L/M0-L. Together, these results revealed that 83.3% (106 genes) of differentially m^6^A-associated genes in M1-L/M0-L were negatively correlated with m^6^A-associated genes in M2-L/M1-L, suggesting that these genes might play an important role in the inflammatory response of microglia.

### Differentially m^6^A-modified mRNAs are linked to several pathophysiological processes

**Fig. 4:**
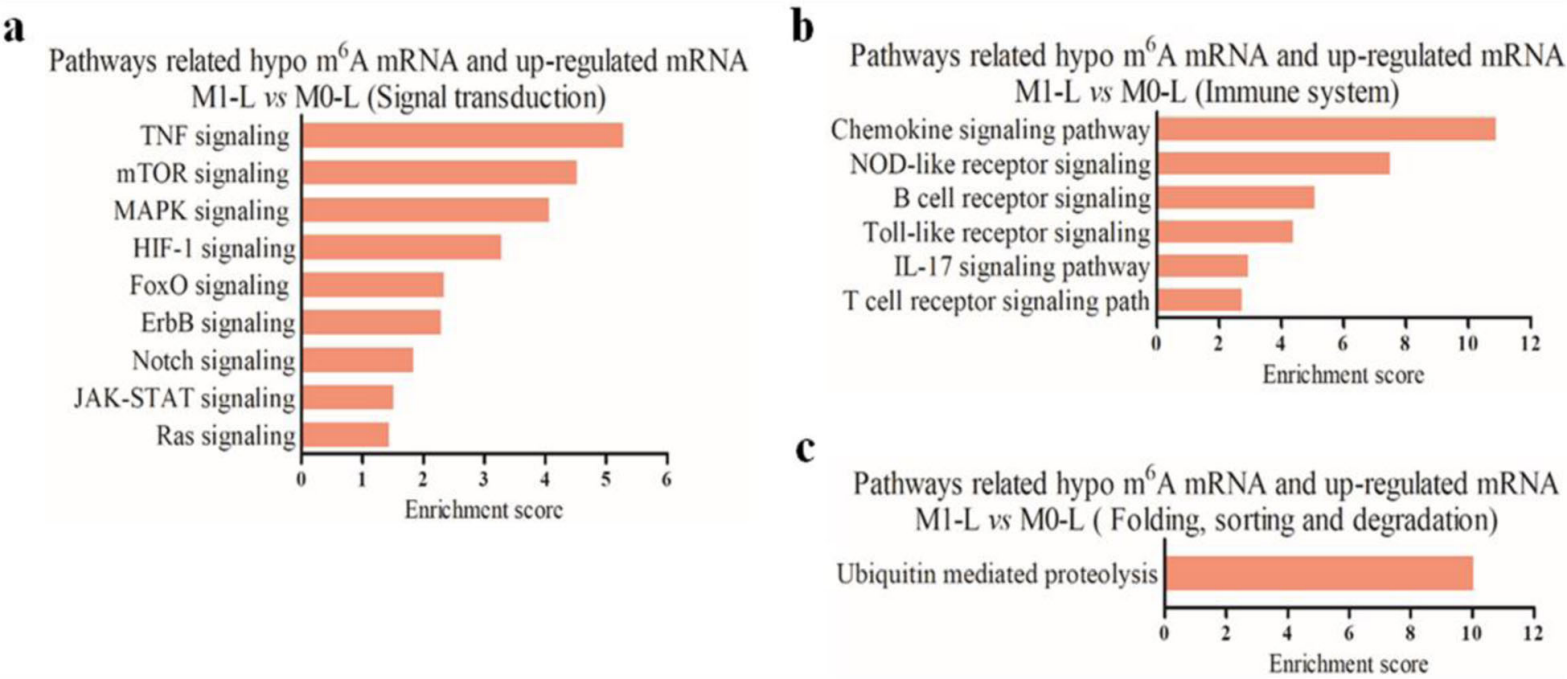
Biological function predictions of the hypo-upregulated mRNAs in M1-L versus M0-L by pathway analysis. **A**–**C** Pathway analysis was applied to 46 upregulated hypo-methylated mRNAs and revealed that 16 upregulated pathways were involved in three biological processes (*P* < 0.05). Enrichment scoring = −log10 (*P* value)

The identification of m^6^A-associated genes is important for exploring the molecular functions underlying m^6^A modification. Firstly, KEGG enrichment analysis of 554 mRNAs (Fig. 2C), which were upregulated in M1-L/M0-L (39 hyper-methylated and 515 hypo-methylated m^6^A modifications) identified 16 significantly upregulated pathways. These pathways were associated with 46 hypo-methylated/upregulated mRNAs (*P* < 0.05, Fig. 4A–C, Additional file 9). The hypo-methylated m^6^A modification patterns were predominantly involved in three major processes including (i) signal transduction (environmental information processing), (ii) immune system processing, and (iii) folding, sorting, and degradation (genetic information processing).

**Fig. 5:**
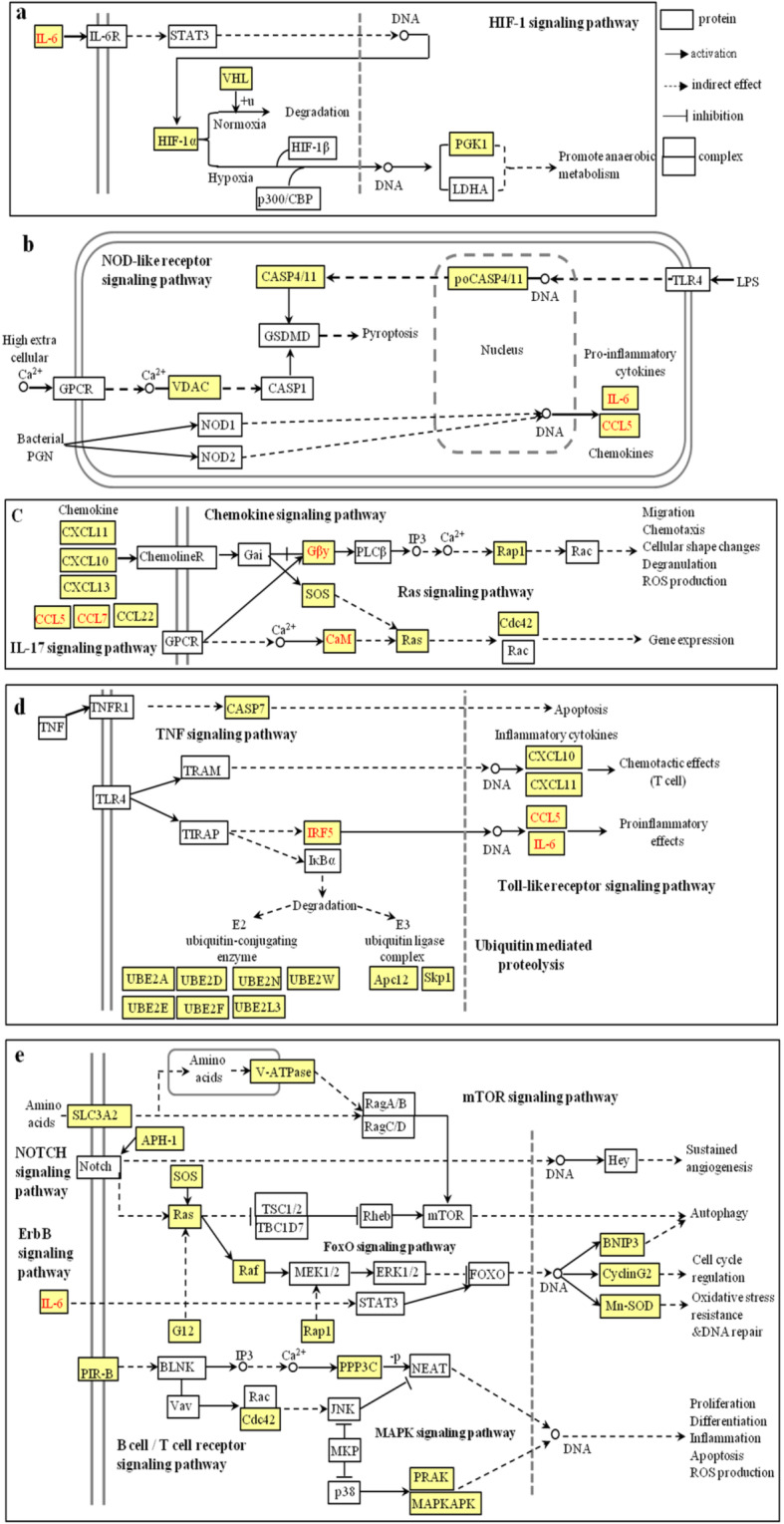
Schematic overviews of signaling pathways associated with 46 upregulated and m^6^A hypo-methylated mRNAs in M1-L/M0-L**.** The yellow squares represent the location of 46 hypo m^6^A and upregulated mRNAs in the related pathways and white squares represent other mRNAs of the pathways which were not be regulated by m^6^A modification. The red letters represent the m^6^A hyper-methylated mRNAs in M2-L/M1-L. Detailed descriptions of the proteins, m^6^A mRNAs, and pathways are presented in Table 1

A total of 46 genes (Table 1) were associated with the HIF-1 signaling pathway (Fig. 5A), the NOD-like receptor signaling pathway (Fig. 5B), the chemokine, IL-17 and Ras signaling pathway (Fig. 5C), the tumor necrosis factor (TNF), ubiquitin-mediated proteolysis, and Toll-like receptor signaling pathways (Fig. 5D), and the mTOR, NOTCH, ErbB, FoxO, B cell/T cell receptor, and MAPK signaling pathways (Fig. 5E).

**Table 1:** Detailed descriptions of the protein and mRNA in the pathways related hypo m^6^A and upregulated mRNA (M1-L *vs* M0-L)

Pathway	Protein	m^6^A mRNA	Protein	m^6^A mRNA	Protein	m^6^A mRNA	Protein	m^6^A mRNA
Chemokine signaling	CCL5	Ccl5	CXCL10	Cxcl10	CXCL13	Cxcl13	CXCL11	Cxcl11
	CCL22	Ccl22	Gβy	Gnb1	SOS	Sos2	Rap1	Rapla
	Gβy	Gng10	Gβy	Gng12	Gβy	Gnb5	CCL7	Ccl7
	Cdc42	Cdc42						
Ubiquitin mediated proteolysis	UBE2D	Ube2d3/2d2	UBE2A	Ube2a	UBE2L3	Ube2l3	UBE2N	Ube2n
	UBE2E	Ube2e1	UBE2F	Ube2f	VHLbox	Vhl	Apc12	Cdc26
	Skp1	Skp1	UBE2W	LOC103694506				
NOD-like receptor signaling	IL6	Il6	CCL5	Ccl5	CASP4	Casp4	VDAC	Vdac1/Vdac3
TNF signaling	IL6	Il6	CCL5	Ccl5	CXCL10	Cxcl10	CASP7	Casp7
B cell receptor signaling	PIR-B	Pir	SOS	Sos2	CAN	Ppp3r1		
mTOR signaling	SOS	Sos2	SLC3A2	Slc3a2	V-ATPase	Atp6v1h		
Toll-like receptor signaling	IL6	Il6	CCL5	Ccl5	CXCL10	Cxcl10	CXCL11	Cxcl11
	IRF5	Irf5						
MAPK signaling	SOS	Sos2	Raf	Araf	G12	Gng12	Ras	Rras2
	Rap1	Rap1a	PRAK	Mapkapk5	PPP3C	Ppp3r1	Cdc42	Cdc42
	MAPKAPK	Mapkapk2						
HIF-1 signaling	IL6	Il6	HIF-1α	Hif1α	PGK1	Pgk1	VHL	Vhl
IL-17 signaling	IL6	Il6	CCL7	Ccl7	CXCL10	Cxcl10		
T cell receptor signaling	SOS	Sos2	PPP3C	Ppp3r1	Cdc42	Cdc42		
FoxO signaling	IL6 CyclinG2	Il6 Ccng2	SOS Mn-SOD	Sos2 Sod2	Raf	Araf	BNIP3	Bnip3
ErbB signaling	Raf	Araf	SOS	Sos2				
Notch signaling	APH-1	LOC108348064						
JAK-STAT signaling	IL6	Il6						
Ras signaling	Cdc42	Cdc42	Ras	Rras2	Gβy	Gnb1	Gβy	Gnb5
	CaM	Calm2	Rap1	Rap1a	Gβy	Gng10	Gβy	Gng12

**Fig. 6:**
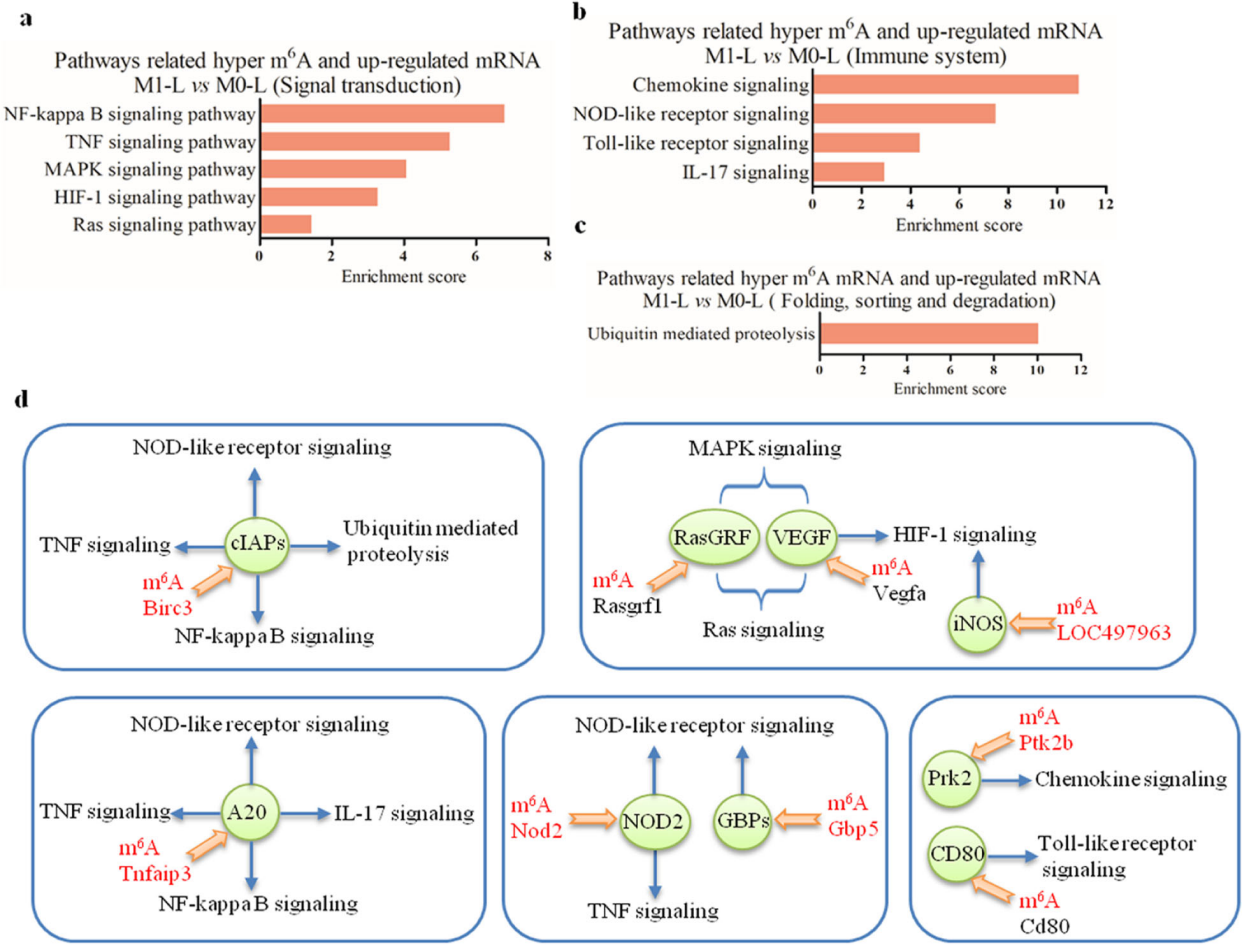
Functional predictions of the hyper-upregulated mRNAs in M1-L versus M0-L based on pathway analysis. **A**–**C** Pathway analysis was applied to 9 mRNAs and revealed that 10 upregulated pathways were involved in 3 biological processes (*P* < 0.05). Enrichment scoring = −log10 (*P* value). **D** Nine m^6^A hyper-methylated mRNAs and their proteins are in the 10 upregulated signaling pathways. The green circles represent proteins, the orange arrows show the mRNA and protein pair, and blue arrows indicate signaling pathways that proteins involved. The mRNAs using red font represent the m^6^A mRNAs that were hypo-methylated in M2-L vs M1-L

Secondly, KEGG analysis revealed that nine upregulated mRNAs with hyper-methylation in M1-L/M0-L were involved in 10 significantly upregulated pathways, including signal transduction, immune system processing, and protein degradation (*P* < 0.05, Fig. 6A–C). These pathways may represent diverse pro-inflammatory processes that can occur during microglial activation. The *Tnfaip3* and *Birc3* genes participated in four signaling pathways, with three of these pathways in common: NOD-like receptor signaling, TNF signaling, and nuclear factor-kappa B (NF-κB) signaling pathways (Fig. 6D). The seven other genes and their associated signaling pathways are shown in Fig. 6D.

**Fig. 7:**
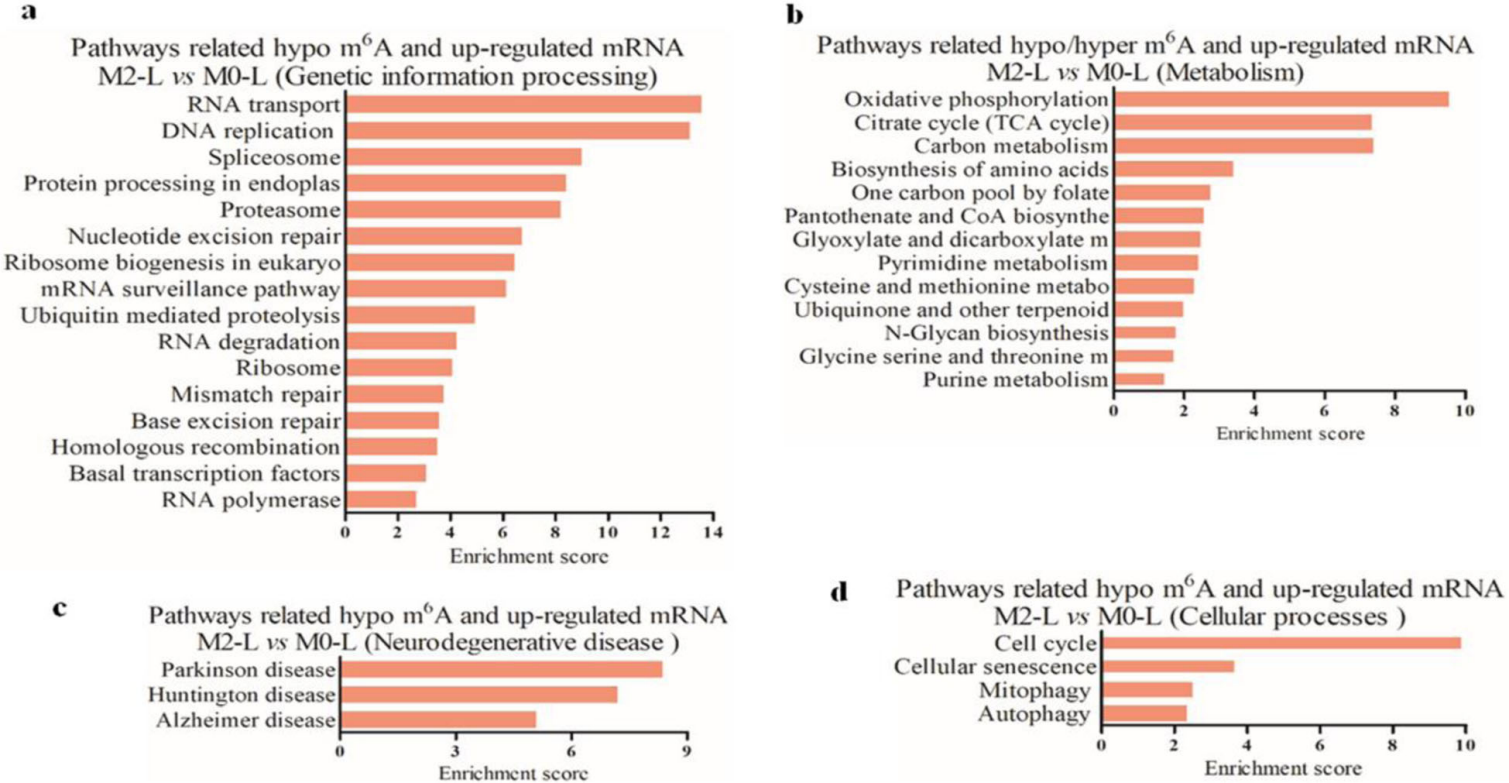
Functional predictions of the upregulated mRNAs with hypo-methylation or hyper-methylation in M2-L versus M0-L based on pathway analysis**.** Pathway analysis was applied to 65 upregulated mRNAs in M2-L/M0-L that were hypo- or hyper-methylated, and revealed that 36 upregulated pathways were involved in four biological processes (*P* < 0.05). Enrichment scoring = −log10 (*P* value)

Finally, KEGG enrichment analysis of the 55 mRNAs, which were upregulated in M2-L/M0-L with distinct m^6^A modification patterns (52 mRNAs were hypo-methylated, and 3 mRNAs were hyper-methylated), identified the following upregulated pathways: genetic information processing, metabolism, cellular processes, and neurodegenerative disease-related pathways (*P* < 0.05, Fig. 7, Tables 2 and 3, see Additional file 10 for further details). Together, our results demonstrate that differentially m^6^A-modified mRNAs are linked to multiple biological processes.

**Table 2:** Detailed descriptions of the genes in the pathways related hypomethylated m^6^A and upregulated mRNA (M2-L vs M0-L)

Gene symbol	Pathway	Gene symbol	Pathway
*Ndufs3*	Oxidative phosphorylation; Alzheimer disease; Parkinson disease; Huntington disease	*Tyms*	One carbon pool by folate; Pyrimidine metabolism
*Tusc3*	Protein processing in endoplasmic reticulum ; N-Glycan biosynthesis	*Rpia*	Carbon metabolism; Biosynthesis of amino acids
*Ch25h*	Primary bile acid biosynthesis	*Exosc8*	RNA degradation
***Pole2***	DNA replication; Nucleotide excision repair; Base excision repair	*Sap18*	RNA transport; mRNA surveillance pathway
*Rps3a*	Ribosome	*Nup35*	RNA transport
*LOC103694506*	Ubiquitin mediated proteolysis	*Rad51c*	Homologous recombination
*Ube2a*	Ubiquitin mediated proteolysis	*Snrpd3*	Spliceosome
*Ero1a*	Protein processing in endoplasmic reticulum	*Srsf10*	Spliceosome
***Psat1***	Carbon metabolism; Biosynthesis of amino acids; Cysteine and methionine metabolism; Glycine, serine and threonine metabolism	*Cdk7*	Cell cycle; Nucleotide excision repair; Basal transcription factors
*Ak6*	Ribosome biogenesis in eukaryotes; Purine metabolism	*Tbp*	Huntington disease; Basal transcription factors
*Lsm8*	Spliceosome; RNA degradation	*Rpl15*	Ribosome
*Cycs*	Parkinson disease; Huntington disease; Alzheimer disease;	*Vkorc1l1*	Ubiquinone and other terpenoid-quinone biosynthesis
*Sec11c*	Protein export	*Rpl26*	Ribosome
*Rpa3*	DNA replication; nucleotide excision repair; mismatch repair; homologous recombination;	*Prps1*	Carbon metabolism; Biosynthesis of amino acids
*Eif2s3y*	RNA transport	*Rbbp4*	Cellular senescence
*Nudt21*	mRNA surveillance pathway	*C1d*	RNA degradation; mitophagy
*Ssr1*	Protein processing in endoplasmic reticulum	*Taf13*	Basal transcription factors
*Snrpb2*	Spliceosome	*Psma3*	Proteasome
*Skp1*	Cell cycle; ubiquitin mediated proteolysis; Protein processing in endoplasmic reticulum	*Polr2k*	Huntington disease; RNA polymerase
***Ndufb11***	Oxidative phosphorylation; Parkinson disease; Huntington disease; Alzheimer disease;	*Lsm5*	Spliceosome; RNA degradation
*Uqcrh*	Oxidative phosphorylation; Alzheimer disease; Parkinson disease; Huntington disease;	*Ppp3r1*	Alzheimer disease; Cellular senescence;
*Ppp2cb*	mRNA surveillance pathway; autophagy; AMPK signaling pathway; Sphingolipid signaling pathway	*Ndufb3*	Oxidative phosphorylation; Parkinson disease; Huntington disease; Alzheimer disease;
*Rps11*	Ribosome	*Mrps21*	Ribosome
*Aasdhppt*	Pantothenate and CoA biosynthesis	*Thoc6*	RNA transport
*Mrpl35*	Ribosome	*Bnip3*	Mitophagy; autophagy
*Mdh1*	Carbon metabolism; citrate cycle (TCA cycle); Glyoxylate and dicarboxylate metabolism; Cysteine and methionine metabolism	***Ccnh***	Cell cycle; Basal transcription factors; Nucleotide excision repair
*Exosc3*	RNA degradation

**Table 3:** Detailed descriptions of the genes in the pathways related hypermethylated m^6^A and upregulated mRNA (M2-L vs M0-L)

Gene symbol	Pathway	Gene symbol	Pathway
***Dpyd***	Pyrimidine metabolism	*Aldh1l1*	One carbon pool by folate;
*Atp6v0d2*	Oxidative phosphorylation		

### Identification of the possible roles of m^6^A-modified lncRNAs based on genomic co-localization relative to differentially expressed mRNAs

To explore the possible roles of differentially m^6^A-modified lncRNAs during microglial activation, 87 m^6^A-associated lncRNAs were selected which were hyper-methylated in M1-L/M0-L and hypo-methylated in M2-L/M1-L. Only 3 m^6^A lncRNAs were hypo-methylated in M1-L/M0-L and hyper-methylated in M2-L/M1-L (Additional file 11). We did not identify any differentially m^6^A-methylated lncRNAs shared between M1-L/M0-L and M2-L/M0-L, which is consistent with the m^6^A methylation of mRNA.

**Fig. 8:**
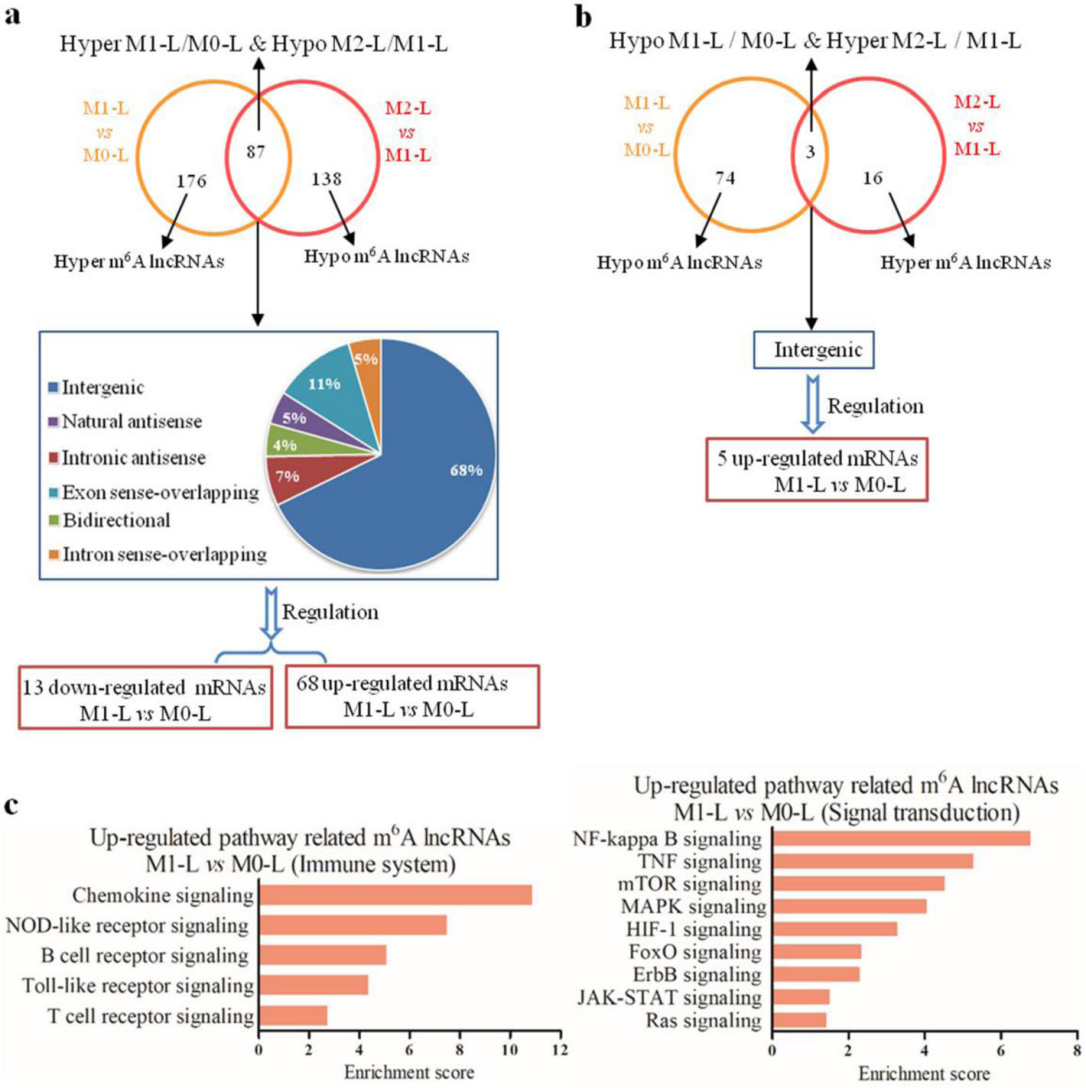
Biological function predictions of the m^6^A lncRNAs based on pathway analysis of their adjacent coding-genes within 300 kb in the genome. **A** Subgroup analysis of 87 altered m^6^A lncRNAs that were hyper-methylated in M1-L/M0-L and hypo-methylated in M2-L/M1-L in relation to their nearby coding genes. **B** Subgroup analysis of three altered m^6^A lncRNAs that were hypo-methylated in M1-L/M0-L and hyper-methylated in M2-L/M1-L in relation to their nearby coding genes. **C** Pathway analysis was applied to 13 mRNAs that were adjacent to 11 m^6^A-modified lncRNAs and revealed that 14 upregulated pathways were associated with two biological processes. Enrichment scoring = −log10 (*P* value)

Next, we analyzed the genomic locations of these 87 lncRNAs and classified them into intergenic (between two coding genes), overlapping exons or introns, natural or intronic antisense, and bidirectional lncRNAs. Our results indicated that most of the differentially m^6^A methylated lncRNAs were intergenic. We combined 87 lncRNAs with their adjacent coding genes (located within 300 kb) and identified differentially expressed genes in M1-L/M0-L to analyze the potential functions of these m^6^A-modified lncRNAs. We found that 13 downregulated and 68 upregulated mRNAs in M1-L/M0-L were associated with 41 lncRNAs (Fig. 8A, Additional file 12). The three m^6^A lncRNAs that were hypo-methylated in M1-L/M0-L and hyper-methylated in M2-L/M1-L were intergenic lncRNAs (Fig. 8B). And, the five upregulated mRNAs in M1-L/M0-L were intergenic gene of these 3 lncRNAs (Additional file 12).

**Fig. 9:**
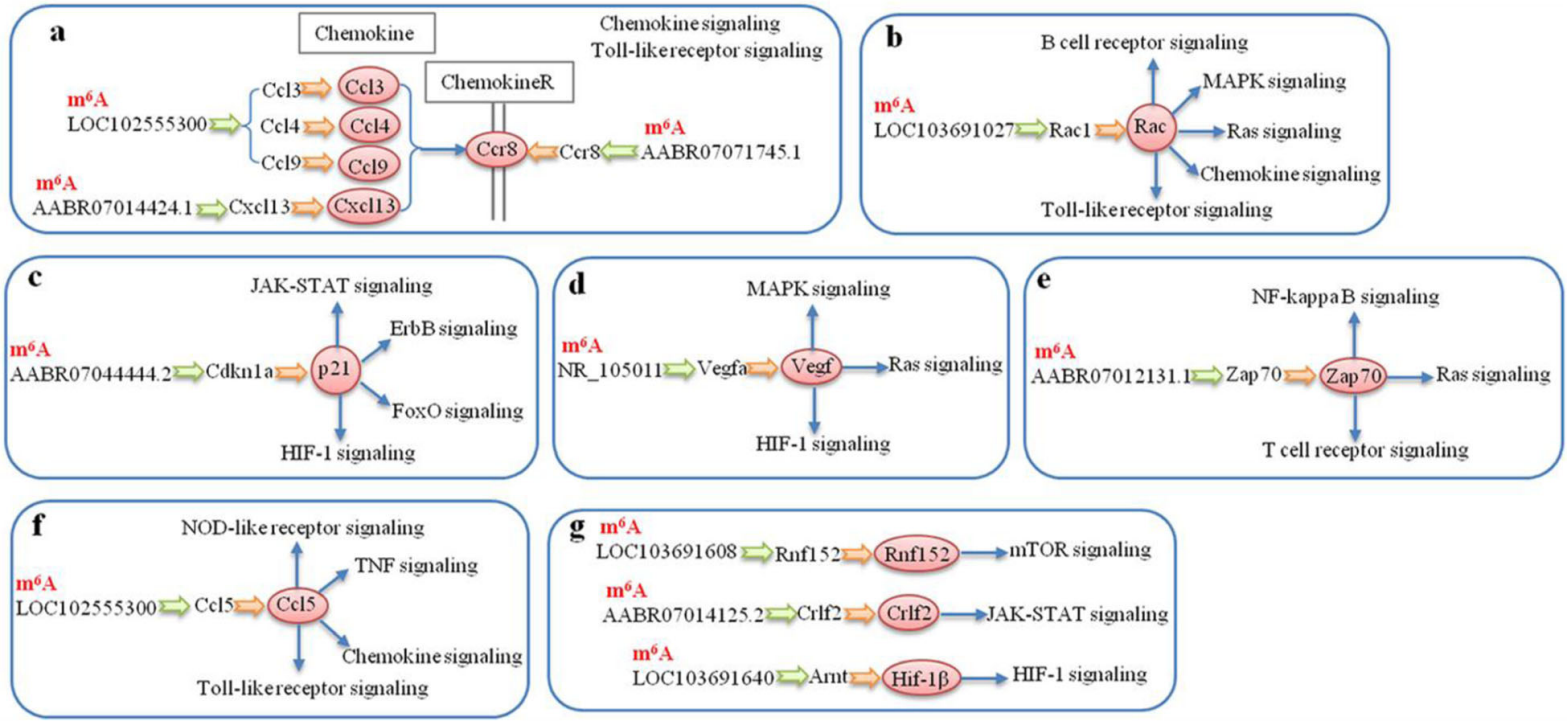
Schematic overviews of the signaling pathways in which 10 m^6^A lncRNAs are probably involved. Ten m^6^A methylated lncRNAs, their regulated mRNAs and proteins are in the 14 upregulated signaling pathways. The red circles represent proteins, the green arrows represent the regulatory relationship of lncRNA to mRNA, the orange arrows show the mRNA and protein pair, and blue arrows indicate signaling pathways that proteins are involved

Furthermore, our results indicate that 11 m^6^A-modified lncRNAs may regulate the expression of the 13 upregulated mRNAs associated with 14 significantly upregulated pathways, including immune system and signal transduction processes (Fig. 8C). These pathways might regulate inflammation, reactive oxygen species production, and pro-inflammatory responses during microglial activation. With the exception of LOC103691608, AABR07014125.2, and LOC103691640, the other eight lncRNAs were involved in the regulation of multiple signaling pathways (Fig. 9).

### Validation of the microarray data by MeRIP and qRT-PCR analyses

**Fig. 10:**
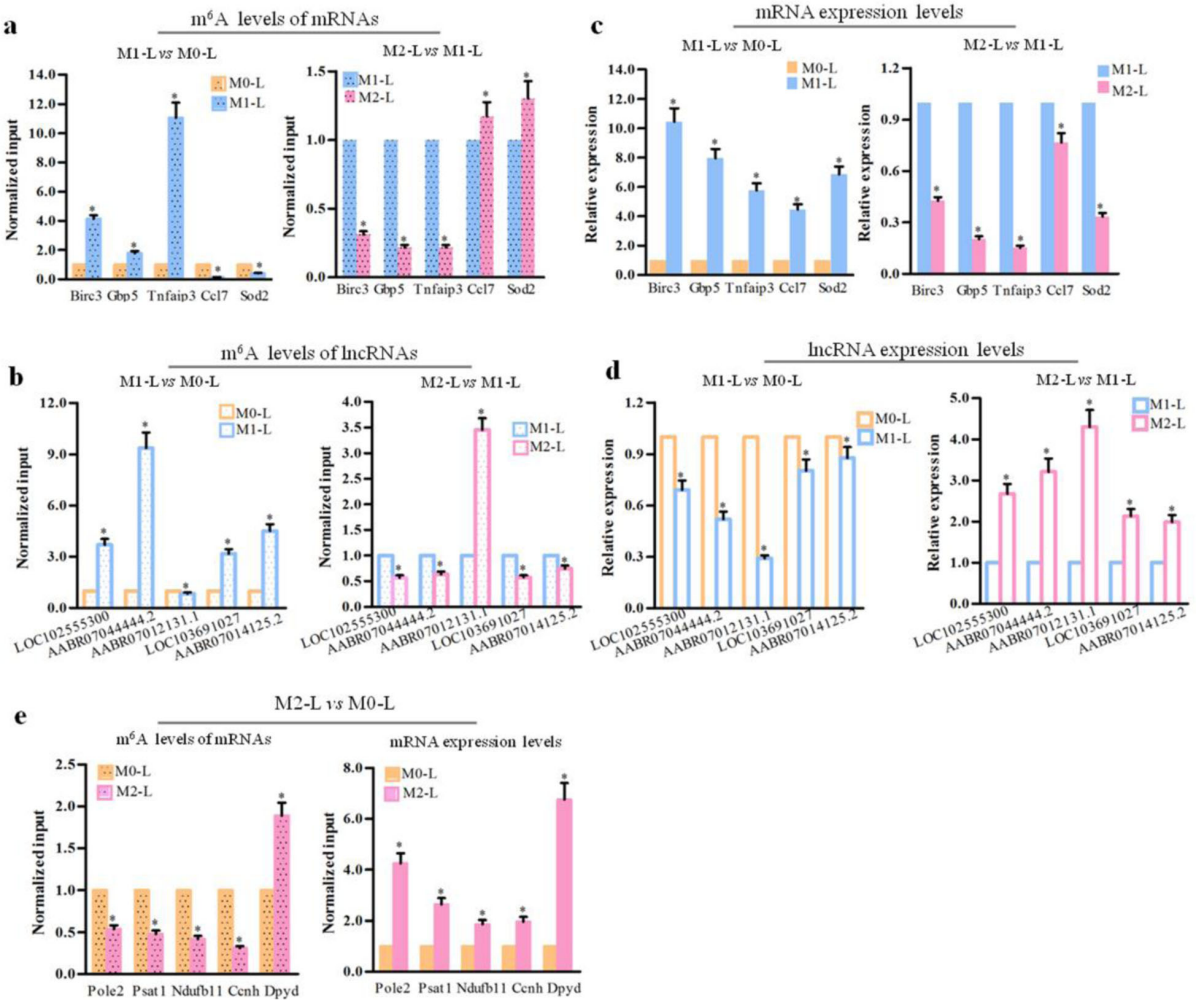
The m^6^A methylation level and expression analysis of the lncRNAs and mRNAs in different phenotypes of primary rat microglia. The m^6^A levels of **A** five mRNAs and **B** five lncRNAs were analyzed by MeRIP-qPCR; the expression level of **C** five mRNAs and **D** five lncRNAs were analyzed using qRT-PCR in M1-L vs M0-L and M2-L vs M1-L. **E** The m^6^A level and expression level of five mRNAs were analyzed by MeRIP-qPCR and qRT-PCR, respectively in M2-L vs M1-L. qRT-PCR was performed using the *GAPDH* gene as an internal control. Error bars represent the standard errors of independent samples. n = 3 per group, *P < 0.05

To validate the m^6^A mRNA and lncRNA microarray analysis results, we first analyzed the m^6^A levels of five mRNAs located in the pathway regulation network (Figs. 5 and 6) by MeRIP in M0-L, M1-L, and M2-L microglia. As shown in Fig. 10A, the m^6^A levels of *Birc3*, *Gbp5*, and *Tnfaip3* mRNAs were upregulated in M1-L/M0-L, but decreased in M2-L/M1-L. The m^6^A levels of *Ccl7* and *Gbp5* mRNAs in M1-L were lower than those in M0-L, but higher in M2-L than M1-L. Next, we analyzed expression levels of these five mRNAs to determine whether mRNA expression was regulated by m^6^A modification during the process of microglial polarization. qRT-PCR analysis revealed that these five mRNAs were upregulated in M1-L/M0-L, but downregulated in M2-L/M1-L. The results indicated that hyper-methylated *Birc3*, *Gbp5*, and *Tnfaip3* displayed increased mRNA stability and expression, while the m^6^A modification was associated with degradation of *Ccl7* and *Gbp5* mRNAs, and reduced their expression levels in M1-L (Fig. 10B). Moreover, we analyzed m^6^A modification and expression levels of *Pole2*, *Psat1*, *Ndufb11*, *Ccnh*, and *Dpyd* mRNAs, which were involved in several upregulated pathways (Fig. 7) in M2-L/M0-L microglia. These findings revealed that *Pole2*, *Psat1*, *Ndufb11*, and *Ccnh* mRNAs were hypo-methylated and upregulated expression, while *Dpyd* mRNA was hyper-methylated and upregulated expression in the M2-L/M0-L phenotype (Fig. 10E).

Finally, based on our previous observations (Fig. 9), we analyzed the m^6^A modification and expression level of five lncRNAs. The LOC102555300, AABR07044444.2, LOC103691027, and AABR07014125.2 were hyper-methylated in M1-L/M0-L microglia, and hypo-methylated in M2-L/M1-L, while the m^6^A level of AABR07012131.1 was downregulated in M1-L/M0-L and upregulated in M2-L /M1-L (Fig. 10C). The expression levels of hyper-methylated LOC102555300, AABR07044444.2, LOC103691027, and AABR07014125.2 in M1-L/M0-L were reduced, indicating that the m^6^A modification made these lncRNAs unstable and prone to degradation. In contrast, significantly increased LOC102555300, AABR07044444.2, LOC103691027, and AABR07014125.2 lncRNA levels were observed in M2-L/M1-L. We found that the hypo-methylated lncRNA, AABR07012131.1, was expressed at a lower level in M1-L/M0-L, but displayed increased expression in M2-L/M1-L (Fig. 10D). Taken together, our data show that the changes were consistent between microarray, MeRIP, and qRT-PCR analyses, and further confirmed the findings of the m^6^A mRNA and lncRNA microarray data.

## Discussion

A multitude of signals received from the central and peripheral nervous systems induce microglial responses towards phenotypes that ultimately may support or harm neuronal health [10, 25]. In particular, in vitro stimulation with LPS and IFNγ promotes the differentiation of activated microglia, the so-called M1 pro-inflammatory phenotype, that typically releases destructive pro-inflammatory mediators. In contrast, IL-4 and IL-10 induce an M2 anti-inflammatory phenotype of microglia that possesses neuroprotective properties [6–9]. Therefore, a strategy reversing the spontaneous M2-L to M1-L phenotypic transition in microglia holds enormous potential for the treatment of CNS diseases.

In this study, we extensively explored the potential m^6^A modification pattern and m^6^A-related signatures of microglia under homeostatic (M0-L), pro-inflammatory (M1-L), and anti-inflammatory (M2-L) conditions using m^6^A-methylated RNA IP combined with rat m^6^A mRNA and lncRNA Epitranscriptomic Microarray analysis. We found that m^6^A methylation was altered in 1588 mRNAs and 340 lncRNAs between the M1-L and M0-L phenotypes, and in 315 mRNAs and 38 lncRNAs between the M2-L and M0-L phenotypes, thus, highlighting the potential role of m^6^A modification of RNAs during the inflammatory response in microglia. Understanding the specific m^6^A-modified transcripts that are associated with microglia under homeostatic, anti-inflammatory, and pro-inflammatory conditions are fundamental first steps in defining the multifaceted nature of microglia, and identifying new therapeutic strategies to modulate inflammatory immune responses in CNS diseases.

m^6^A modifications are reversible and executed by methyltransferases (i.e., writers) and demethylases (i.e., erasers), which can be recognized by the m^6^A binding proteins (i.e., readers) to regulate RNA fate [26, 27]. Emerging evidence suggests that m^6^A modification and its regulators play important roles in many neurological diseases such as Alzheimer’s disease, Parkinson’s disease, and stroke [27]. In the present study, we showed that m^6^A modifications in multiple pro- and anti-inflammatory cytokines were remarkably changed in the activated primary microglia, serving as an underlying mechanism to change the mRNA levels of these cytokines. Interestingly, a few pro-inflammatory (e.g., CCL7) and anti-inflammatory cytokines (e.g., A20 and Gbp5) were increased simultaneously in M1-L microglia, which appears to be paradoxical. However, inflammatory responses in tissue repair are the perturbation of the inflammatory homeostasis; therefore, it is the balance of the pro- and anti-inflammatory cytokines that determines the fate of cells and tissues, which, according to our study, are underlined by the balance in m^6^A modifications. Recent reports showed that m^6^A regulators were involved in inflammation of a mouse microglial cell line [28, 29]. Thus, the underlying mechanisms of m^6^A modification during microglia-mediated inflammation need to be further investigated.

This study not only confirms many previously identified microglial signature genes, but also extends these findings to m^6^A methylation status. For example, M1-L showed a dramatic decrease in the expression levels of the homeostatic markers with the hypo-methylated m^6^A modification, such as *Aif1l*, *Gpr34*, and *Gabbr1*. Consistent with a previous study [30], the expression levels of LPS-regulated genes, *Sygn*, *Ccl5*, *Ifitm3*, *Rnf149*, *Sod2*, *Cxcl10*, and *Ch25h*, were significantly increased, but had hypo-methylated m^6^A. In contrast, expression of *Irak3* and *Zbp1* genes were increased and displayed hyper-methylated m^6^A. LPS-induced downregulation of *Ifitm2*, *Cd244*, and *Slfn5* was associated with both hypo-methylation (*Ifitm2*) and hyper-methylation (*Cd244*, *Slfn5*).

In addition, the microglia-enriched receptors, *Ly96*, *Clec4a2*, and *Cd80*, and the microglia-enriched ligand, *B2m*, showed increased expression in M1-L/M0-L, with both hypo-methylation (Ly96, Clec4a2, and B2m) and hyper-methylation (Cd80). Conversely, the microglia-enriched receptors Cd84, Gpr34, Cr1l, and Cfh) were expressed at reduced levels in M1-L, with m^6^A hyper-methylation of Cd84 and Cr1l and m^6^A hypo-methylation of Gpr34 and Cfh. Thus, m^6^A methylation has a dynamic and complex relationship with gene expression in regulating the inflammatory response of microglia.

KEGG analysis showed that pathways known to modulate immune system processes (Ras, HIF-1, TNF, mTOR, Notch, ErbB, FoxO, JAK-STAT, and MAPK signaling), and those involved in signal transduction processes (Chemokine, Ras, NOD-like/Toll-like receptor, B cell/T cell receptor, IL-17 signaling) were the major pathways associated with the differentially m^6^A-methylated mRNAs in M1-L microglia. The Ubiquitin-mediated proteolysis pathway was also activated in the M1-L phenotype. The ubiquitin system regulates cell differentiation and immunity and is involved in transcription, regulation of secretion, and cell development through mediating protein degradation. Our findings were consistent with the resulting “activated” microglia, which exhibit migratory, proliferative, and phagocytic properties, as well as the capacity to release chemokines, cytokines, and neurotrophic factors and to present antigens [4]. In contrast, genetic information processing, metabolism and cellular processes, and neurodegenerative disease-related pathways were the major pathways associated with differentially m^6^A-methylated mRNAs in M2-L microglia. These pathways may fulfill energy and biosynthetic requirements during microglial anti-inflammatory processes.

To the best of our knowledge, this study is the first to reveal the m^6^A methylation profile of lncRNA in M0-L, M1-L and M2-L microglia. We observed differential m^6^A modification of 87 lncRNAs, which were hyper-methylated in M1-L and hypo-methylated in M2-L, as well as three lncRNAs that were hypo-methylated in the M1-L phenotype and hyper-methylated in M2-L. These differentially methylated lncRNAs may modulate inflammatory reaction in microglia by altering various immune system and signal transduction processes, including 14 upregulated KEGG pathways.

In conclusion, our study identified multiple differential m^6^A modifications of mRNAs and lncRNAs among the M0-L, M1-L, and M2-L microglia, and explored the potential roles of these m^6^A modifications associated with pro-inflammatory and anti-inflammatory microglia. Our data suggest that m^6^A modifications of transcripts could serve as a useful regulator during the microglial immune response.

## Supplementary Information

**Additional file 1:.** Primers of mRNAs and lncRNAs for MeRIP

**Additional file 2:.** Primers of mRNAs and lncRNAs for qRT-PCR

**Additional file 3:.** The m^6^A methylation levels of mRNAs differentially expressed between M1-L and M0-L, M2-L and M1-L

**Additional file 4:.** The m^6^A methylation level of lncRNAs differentially expressed between M1-L and M0-L0, M2-L and M1-L

**Additional file 5:.** The mRNA expression level differentially expressed between M1-L and M0-L, M2-L and M1-L

**Additional file 6:.** The associated analysis of mRNA expression level and methylation level between M1-L and M0-L

**Additional file 7:.** The associated analysis of mRNA expression level and methylation level between M2-L and M0-L

**Additional file 8:.** The associated analysis of mRNA expression level and methylation level between M2-L and M1-L

**Additional file 9:.** The up-regulated mRNAs related pathways between M1-L and M0-L

**Additional file 10:.** The up-regulated mRNAs related pathways between M2-L and M0-L

**Additional file 11:.** The 87 m^6^A lncRNAs of hyper methylation M1-L vs M0-L and hypo methylation M2-L vs M1-L

**Additional file 12:.** The 3 m^6^A lncRNAs of hypo methylation M1-L vs M0-L and hyper methylation M2-L vs M1-L

**Additional file 13:.** The mRNAs differentially expressed between M1-L and M0-L, M2-L and M1-L, and related 43 lncRNAs

## Data Availability

All data generated or analyzed during this study are included in the published article or supplementary files.

## Abbreviations

CNS: Central nervous system
IFN-γ: Interferon-γ
IL: Interleukin
KEGG: Kyoto Encyclopedia of Genes and Genomes
lncRNAs: Long non-coding RNAs
LPS: Lipopolysaccharide
M0-L: Rest phenotype unstimulated
M1-L: Pro-inflammatory M1-like phenotype
M2-L: Anti-inflammatory M2-like phenotype
m^6^A: N6-methyladenosine
MeRIP: Methylated RNA immunoprecipitation
NF-κB: Nuclear factor-kappa B
qRT-PCR: Quantitative real-time polymerase chain reaction
TNF: Tumor necrosis factor

## References

[1] Lawson LJ, Perry VH, Dri P, Gordon S (1990). Heterogeneity in the distribution and morphology of microglia in the normal adult mouse brain. Neuroscience..

[2] Axel N, Frank K, Fritjof H (2005). Resting microglial cells are highly dynamic surveillants of brain parenchyma in vivo. Science..

[3] Katsumoto A, Lu H, Miranda AS, Ransohoff RM (2014). Ontogeny and functions of central nervous system macrophages. J Immunol..

[4] Prinz M, Priller J (2014). Microglia and brain macrophages in the molecular age: from origin to neuropsychiatric disease. Nat Rev Neurosci..

[5] Gautier EL, Shay T, Miller J, Greter M, Jakubzick C, Ivanov S (2012). Gene expression profiles and transcriptional regulatory pathways that underlie the identity and diversity of mouse tissue macrophages. Nat Immunol..

[6] Beutner C, Linnartz-Gerlach B, Schmidt SV, Beyer M, Mallmann MR, Staratschek-Jox A, Schultze JL, Neumann H (2013). Unique transcriptome signature of mouse microglia. Glia..

[7] Butovsky O, Jedrychowski MP, Moore CS, Cialic R, Lanser AJ, Gabriely G, Koeglsperger T, Dake B, Wu PM, Doykan CE, Fanek Z, Liu LP, Chen Z, Rothstein JD, Ransohoff RM, Gygi SP, Antel JP, Weiner HL (2014). Identification of a unique TGF-beta-dependent molecular and functional signature in microglia. Nat Neurosci..

[8] Kigerl KA, Gensel JC, Ankeny DP, Alexander JK, Donnelly DJ, Popovich PG (2009). Identification of two distinct macrophage subsets with divergent effects causing either neurotoxicity or regeneration in the injured mouse spinal cord. J Neurosci..

[9] Durafourt BA, Moore CS, Zammit DA, Johnson TA, Zaguia F, Guiot MC, Bar-Or A, Antel JP (2012). Comparison of polarization properties of human adult microglia and blood-derived macrophages. Glia..

[10] Crotti A, Ransohoff RM (2016). Microglial physiology and pathophysiology: insights from genome-wide transcriptional profiling. Immunity..

[11] Hickman SE, Kingery ND, Ohsumi TK, Borowsky ML, Wang LC, Means TK, el Khoury J (2013). The microglial sensome revealed by direct RNA sequencing. Nat Neurosci..

[12] Zhang Y, Chen K, Sloan SA, Bennett ML, Scholze AR, O’Keeffe S (2014). An RNA-sequencing transcriptome and splicing database of glia, neurons, and vascular cells of the cerebral cortex. J Neurosci..

[13] Lewis ND, Hill JD, Juchem KW, Stefanopoulos DE, Modis LK (2014). RNA sequencing of microglia and monocyte-derived macrophages from mice with experimental autoimmune encephalomyelitis illustrates a changing phenotype with disease course. J Neuroimmunol..

[14] Olah M, Amor S, Brouwer N, Vinet J, Eggen B, Biber K, Boddeke HWGM (2012). Identification of a microglia phenotype supportive of remyelination. Glia..

[15] Verheijden S, Beckers L, Casazza A, Butovsky O, Mazzone M, Baes M (2015). Identification of a chronic non-neurodegenerative microglia activation state in a mouse model of peroxisomal beta-oxidation deficiency. Glia..

[16] Keren-Shaul H, Spinrad A, Weiner A, Matcovitch-Natan O, Dvir-Szternfeld R, Ulland TK, David E, Baruch K, Lara-Astaiso D, Toth B, Itzkovitz S, Colonna M, Schwartz M, Amit I (2017). A unique microglia type associated with restricting development of Alzheimer’s disease. Cell..

[17] Orre M, Kamphuis W, Osborn LM, Jansen AHP, Kooijman L, Bossers K, Hol EM (2014). Isolation of glia from Alzheimer’s mice reveals inflammation and dysfunction. Neurobiol Aging..

[18] Sousa C, Golebiewska A, Poovathingal KS, Kaoma T, Pires-Afonso Y, Martina S, et al. Single-cell transcriptomics reveals distinct inflammation-induced microglia signatures. EMBO Rep. 2018;19(11):e46171. doi: 10.15252/embr.201846171. 10.15252/embr.201846171 PMC6216255 30206190

[19] Heinz S, Benner C, Spann N, Bertolino E, Lin YC, Laslo P, Cheng JX, Murre C, Singh H, Glass CK (2010). Simple combinations of lineagedetermining transcription factors prime cis-regulatory elements required for macrophage and B cell identities. Mol Cell..

[20] Lavin Y, Winter D, Blecher-Gonen R, David E, Keren-Shaul H, Merad M, Jung S, Amit I (2014). Tissue-resident macrophage enhancer landscapes are shaped by the local microenvironment. Cell..

[21] Alarcón CR, Lee H, Goodarzi H, Halberg N, Tavazoie SF (2015). N6-methyladenosine marks primary microRNAs for processing. Nature..

[22] Patil DP, Chen CK, Pickering BF, Chow A, Jackson C, Guttman M, Jaffrey SR (2016). m^6^A RNA methylation promotes XIST-mediated transcriptional repression. Nature..

[23] Zhao BS, Roundtree IA, He C (2017). Post-transcriptional gene regulation by mRNA modifications. Nat Rev Mol Cell Biol..

[24] Losciuto S, Dorban G, Gabel S, Gustin A, Hoenen C, Grandbarbe L, Heuschling P, Heurtaux T (2012). An efficient method to limit microglia-dependent effects in astroglial cultures. J Neurosci Methods..

[25] Sousa C, Biber K, Michelucci A (2017). Cellular and molecular characterization of microglia: a unique immune cell population. Front Immunol..

[26] Shulman Z, Stern-Ginossar N (2020). The RNA modification N 6-methyladenosine as a novel regulator of the immune system. Nat Immunol..

[27] Chokkalla AK, Mehta SL, Vemuganti R (2020). Epitranscriptomic regulation by m(6)A RNA methylation in brain development and diseases. J Cereb Blood Flow Metab..

[28] Wen L, Sun W, Xia D, Wang Y, Li J, Yang S. The m6A methyltransferase METTL3 promotes LPS-induced microglia inflammation through TRAF6/NF-kappaB pathway. Neuroreport. 2020. Online ahead of print. 10.1097/WNR.0000000000001550. 10.1097/WNR.0000000000001550 33165191

[29] Zheng L, Tang X, Lu M, Sun S, Xie S, Cai J (2020). microRNA-421-3p prevents inflammatory response in cerebral ischemia/reperfusion injury through targeting m^6^A Reader YTHDF1 to inhibit p65 mRNA translation. Int Immunopharmacol.

[30] Bennett ML, Bennett FC, Liddelow SA, Ajami B, Zamanian JL, Fernhoff NB, Mulinyawe SB, Bohlen CJ, Adil A, Tucker A, Weissman IL, Chang EF, Li G, Grant GA, Hayden Gephart MG, Barres BA (2016). New tools for studying microglia in the mouse and human CNS. Proc Natl Acad Sci U S A..

