# Supplementary material for: The potential roles of m6A modification in regulating the inflammatory response in microglia
Source: J Neuroinflammation. 2021 Jul 5;18:149. doi: 10.1186/s12974-021-02205-z (PMC8259013; doi:10.1186/s12974-021-02205-z)
Supplement: Supplementary file 1 — Additional file 1:. Primers of mRNAs and lncRNAs for MeRIP [file 12974_2021_2205_MOESM1_ESM.doc]

| **Gene Symbol** | **Transcript ID** | **Sequence (5'-3')** |
| --- | --- | --- |
| Birc3 | NM_023987 | F: TCTGAGGAAGTGTCCCATCTGC  R: ACCATCAGTCCAGTCCTGTTCA |
| Gbp5 | ENSRNOT00000037339 | F: ACCGAAGGACTTCAGACAACCA  R: GCCCGCACCCAGTTGAGAAA |
| Tnfaip3 | ENSRNOT00000074583 | F: TGTCGCTGCTCCACCTACCA  R: CGCTCTGCCGATGTCCGTTT |
| Ccl7 | ENSRNOT00000000256 | F: CCTGAAGACAGATGCCTGAACAGAA  R: GTAGTCCACCCATTTCAGCACAGTT |
| Sod2 | ENSRNOT00000025794 | F: GCTAGGCTCCTGACTGACAACT  R: GCTGAATGGCTTCCCTGAATGC |
| LOC102555300 | XR_338758 | F: TGTCTGCTGAGTGGACTGGACTATA  R: GATTCGGTCGAAGTGGACCTGTT |
| AABR07044444.2 | ENSRNOT00000092596 | F: AGAACGCTAACACCACGGACATC  R: CCTCTGCTCTGACGAACCCTTG |
| AABR07012131.1 | ENSRNOT00000093682 | F: TGGGACAGAGGAGATGGCAGATAA  R: CTTGTGGAGGTCAGAGGTGAACTTG |
| LOC103691027 | XR_590053 | F: CCAGGATCGTCGTCCAGTGAGT  R: CGAAGATGTGACTTGAGTCTCCAGG |
| AABR07014125.2 | ENSRNOT00000084593 | F: TGGCTCCAGAGAGGTCACACA  R: AGACTGTCACGGAATTAGAAACTGA |
| Pole2 | ENSRNOT00000005835 | F: GGTGACGTGATTGTCCTTGGAATGA  R: GTGTATAGGCCACTGTGGAACTGAG |
| Psat1 | NM_198738 | F: CCGTGAGCAGCGTTCAGAGTAA  R: GTAGGAGTGAAGGTGTCCGTTAGC |
| Ndufb11 | ENSRNOT00000011183 | F: TGGAATATGCGAGCTGTCTTCTTCT  R: CTGGTAACTGGATCTTGCTGGGAT |
| Ccnh | ENSRNOT00000049423 | F: GACAGGAGAAGGCACTGGAACA  R: ATGGATTGTGGACAATGAGGTGAA |
| Dpyd | ENSRNOT00000055723 | F: TGCCTCAGCGTCTGCCCTATT  R: CGGCTTCACGGCTAATGGTAGG |

Table S1 Primers of mRNAs and lncRNAs for MeRIP
