# Supplementary material for: The potential roles of m6A modification in regulating the inflammatory response in microglia
Source: J Neuroinflammation. 2021 Jul 5;18:149. doi: 10.1186/s12974-021-02205-z (PMC8259013; doi:10.1186/s12974-021-02205-z)
Supplement: Supplementary file 2 — Additional file 2:. Primers of mRNAs and lncRNAs for qRT-PCR [file 12974_2021_2205_MOESM2_ESM.doc]

| **Gene Symbol** | **Transcript ID** | **Sequence (5'-3')** |
| --- | --- | --- |
| Birc3 | NM_023987 | F: CCGAGGAGGAGGAGTCAGATGA  R: GGATTGGTGTCACAGGCGTCAA |
| Gbp5 | ENSRNOT00000037339 | F: GGCTGGGAAGGAGAAAGGCTTT  R: ACGAAGGTGCTACTGAGGAGGA |
| Tnfaip3 | ENSRNOT00000074583 | F: GGTAAGTGCCAAGCCTGCCTTC  R: TGTCTGCTGGAGTCCGTGATGT |
| Ccl7 | ENSRNOT00000000256 | F: GACCAATTCATCCACTTGCTGCTAT  R: GCCTCCTCAACCCACTTCTGAT |
| Sod2 | ENSRNOT00000025794 | F: AAGGAGAGTTGCTGGAGGCTATCA  R: ATTAGAGCAGGCGGCAATCTGTAAG |
| LOC102555300 | XR_338758 | F: ACCAGAAGCTAAGTCACACCTAACC  R: GAGCAGAGTCACTGTCCTGTAGC |
| AABR07044444.2 | ENSRNOT00000092596 | F: CCCACTTCCCACACACGGTCTT  R: ACTGCGTTGTCCTCTGCTCTGA |
| AABR07012131.1 | ENSRNOT00000093682 | F: GGTGGAGGCTAGAACTGCTTGG  R: TGCGGATGCGGATGTGGATG |
| LOC103691027 | XR_590053 | F: GCAAGAGTACACCTGAGCTTCTCG  R: CCAGTAGTCCCGCCGTCTTACT |
| AABR07014125.2 | ENSRNOT00000084593 | F: GGAGTCAGTGAGTCCTGGAAGC  R: AACCTGCCTTTGCTGTGGATGA |
| Pole2 | ENSRNOT00000005835 | F: TGTCCAGGAGTGCAGTCAGTCT  R: CTCGGCTTTGTCCCTTGCTGTT |
| Psat1 | NM_198738 | F: CGCCAGAATAGAAGCAGGATGAACA  R: AGCATTATACAGAGACGCTCGGATG |
| Ndufb11 | ENSRNOT00000011183 | F: TGTATGCTCGCCGCCTGTCA  R: GGTTCTCTTTGCCGCTTTCTCTCC |
| Ccnh | ENSRNOT00000049423 | F: AGTTGGAGCGATGTCATTCTTCTGA  R: CGTCCACTCCTCCTCTTCCTGTT |
| Dpyd | ENSRNOT00000055723 | F: TCATTCATCACAAGTATCGCCAACA  R: ACCATTCCGCAAGTAAGACCAAGA |

Table S2 Primers of mRNAs and lncRNAs for qRT-PCR
